# 2-(1,3-Oxazolin-2-yl)pyridine and 2,6-bis(1,3-oxazolin-2-yl) pyridine

**DOI:** 10.1016/j.dib.2018.09.129

**Published:** 2018-10-04

**Authors:** Wioletta Ochędzan-Siodłak, Anna Bihun-Kisiel, Dawid Siodłak, Anna Poliwoda, Błażej Dziuk

**Affiliations:** Faculty of Chemistry, University of Opole, Oleska 48, 45-052 Opole, Poland

**Keywords:** Ligands, Oxazoline, Pyridine, Conformation, Association

## Abstract

The data presented in this article are related to research articles “Titanium and vanadium catalysts with oxazoline ligands for ethylene-norbornene (co)polymerization (Ochędzan-Siodłak et al., 2018). For the title compounds, 2-(1,3-oxazolin-2-yl)pyridine (Py-ox) and 2,6-bis(1,3-oxazolin-2-yl)pyridine (Py-box), the single-crystal X-ray diffraction measurement together with NMR, GC, MS, DSC analysis, like also the method of crystallization are presented.

**Specifications table**

**Table:** 

Subject area	*Chemistry*
More specific subject area	*Organic Chemistry, Ligands for Catalysts*
Type of data	*Figures, tables, text file.*
	*X-ray (table, figures), GC–MS (Figures)*, 13*C NMR (figures), DSC (figures), synthesis (text)*
How data was acquired	*X-ray (Xcalibur diffractometer),*
	*NMR (Bruker Ultrashield spectrometer 400 MHz, solvent DMSO-d6),*
	*GC–MS (Hewlett Packard HP7890 A GC system)*
	*DSC (2010 TA calorimeter)*
Data format	*X-ray (analyzed), GC–MS (raw), NMR (raw), DSC (raw)*
Experimental factors	*Crystallization at room temperature. Py-ox - highly anhydrous toluene/hexane mixture, Py-box - DMSO-d6 in NMR tube.*
Experimental features	*Highly anhydrous condition for crystals are required.*
Data source location	*City: Opole, Country: Poland, Latitude: N 50*°*40*′*23.981*″*, Longitude: E 17*°*55*′*53.173*′*, (Lat,Long: 50.673328, 17.93143699999996),*
Data accessibility	*The Cambridge Crystallographic Data Centre no. CCDC 1815355 and CCDC 1580983* (http://www.ccdc.cam.ac.uk/conts/retrieving.html, email:deposit@ccdc.cam.ac.uk.).

**Value of the data**

• X-Ray structural information for Py-ox and Py-box compounds not coordinated by metal atom is presented.
• Conformation and association pattern in the crystal state is shown.
• Crystallization methods are shown.
• Purification for Py-ox is improved.

## 1 Data

The presented compounds, 2-(1,3-oxazolin-2-yl)pyridine (Py-ox) and 2,6-bis(1,3-oxazolin-2-yl)pyridine (Py-box), are commonly applied as ligands for complexes with transition metals: cobalt [2], rhenium [3], platinum and palladium [4], [5] for Py-ox, as well as copper [6], [7], ruthenium [8], [9], [10], [11], rhodium [12], manganese [13], silver [14], nickel [15], cobalt [16], terbium [17], and iron [18], in the case of Py-box. Some of them reveal catalytic properties. In our work, the Py-ox and Py-box compounds were applied as ligands for titanium and vanadium complexes, which turned out to be active in polymerization of ethylene and copolymerization of ethylene with norbornene [1]. The X-Ray information for Py-ox and Py-box compounds can be important for comparative studies, to show differences between these compounds not coordinated by metal atom and applied as ligands. It can help to understand dependence between the structure and activity of the designed complexes. The presented crystallization methods are worth to notice. The improved method of purification enable to obtain the studied compound of high quality.

## 2 Experimental design, materials and methods

### 2.1 Synthesis

#### 2.1.1 2-(1,3-oxazolin-2-yl)pyridine (Py-ox)

The synthesis was performed mainly according to Stokes et al. [19]. The crude product was subjected to flash chromatography using the MeOH: AcOEt (1:4) mixture as eluent. Yield 60%. Elemental analysis C_8_H_8_N_2_O results: calculated C 64.85%, H 5.44%, N 18.91%, experimental C 64.92%, H 5.45%, N 19.09%. ^1^H NMR (400 MHz, DMSO-*d6*) δ 8.65 (1H, *J* = 4.5 Hz, d), 7.99 (1H, *J* = 8.0 Hz, d), 7.93 (1H, *J* = 7.8 Hz, td), 7.54 (1H, m), 4.45 (2H, *J* = 9.6 Hz, t), 4.00 (2H, *J* = 9.6 Hz, t). ^13^C NMR (400 MHz, DMSO-*d6*) δ 162.98, 149.53, 146.52, 137.09, 125.90, 123.80, 67.66, 54.61. GC–MS M^+^ 148 m/e. Melting temperature 57.0 (54.6–60.0) °C.

#### 2.1.2 2,6-bis(1,3-oxazolin-2-yl)pyridine (Py-box)

The synthesis was performed mainly according to Zhu et al. [20]. Yield 76%. Elemental analysis C_11_H_11_N_3_O_2_ results: calculated C 64.82%, H 5.10%, N 19.34%, experimental: C 64.88%, H 5.12%, N 19.39%. ^1^H NMR (400 MHz, DMSO-*d6*) δ 8.11 (2H, *J*_*1*_ = 1.2 Hz, *J*_*2*_ = 7.2 Hz, t), 8.02(1H, *J*_*1*_ = 6.4 Hz, *J*_*2*_ = 2.4 Hz, q), 4.45 (4H, *J* = 9.6 Hz, t), 4.01 (4H, *J* = 9.6 Hz, t). ^13^C NMR (400 MHz, DMSO-*d6*) δ 163.10, 147.01, 138.46, 126.00, 68.28, 55.13. GC–MS M^+^ 217 m/e. Melting temperature 160.6 (159.4–163.0) °C.

### 2.2 Crystallization

#### 2.2.1 2-(1,3-oxazolin-2-yl)pyridine (Py-ox)

The crystals were obtained at room temperature from highly anhydrous toluene/hexane mixture. The solvents were freshly distilled over sodium. The highly anhydrous conditions are crucial. All operations were performed in a glove-box filled with argon. Py-ox (20 mg) was placed in a 5 ml snap cap vial with plastic cap and dissolved in toluene (1 ml). Then, hexane (1 ml) was added and the solution was left to stand at room temperature for a week.

#### 2.2.2 2,6-bis(1,3-oxazolin-2-yl)pyridine (Py-box)

The crystals of appropriate quality were obtained at room temperature from DMSO-d6 solution by long standing time in NMR tube. All operations were performed in a glove-box filled with argon. DMSO-d6 solvent from sealed glass ampoules was applied. Py-box (15 mg) and DMSO-d6 (0.6 ml) was placed in NMR tube and the cap was sealed by a parafilm. The solution was left to stand at room temperature for a month.

### 2.3 X-ray

**Fig. 1:**
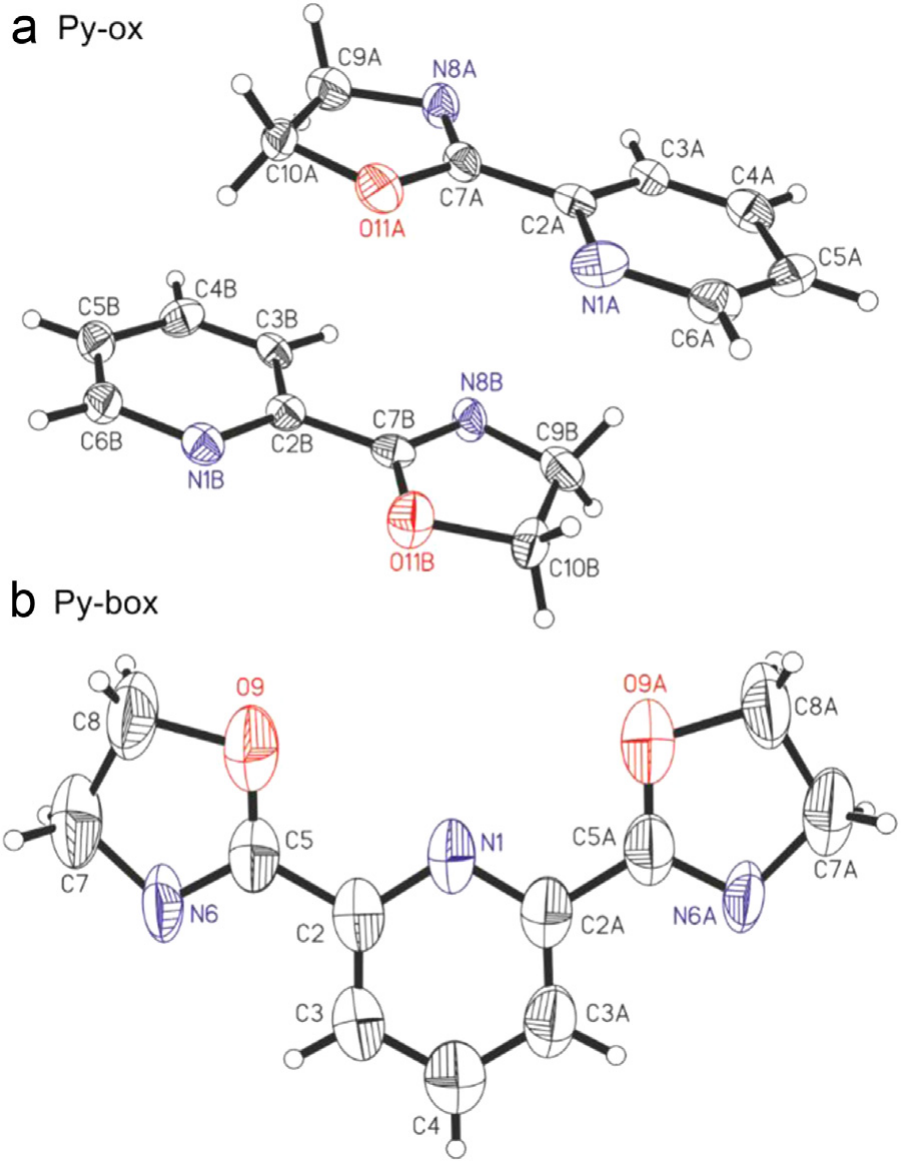
Molecular conformation of Py-ox (a) and Py-box (b) with atom labeling and the displacement ellipsoids at 50% probability level.

**Fig. 2:**
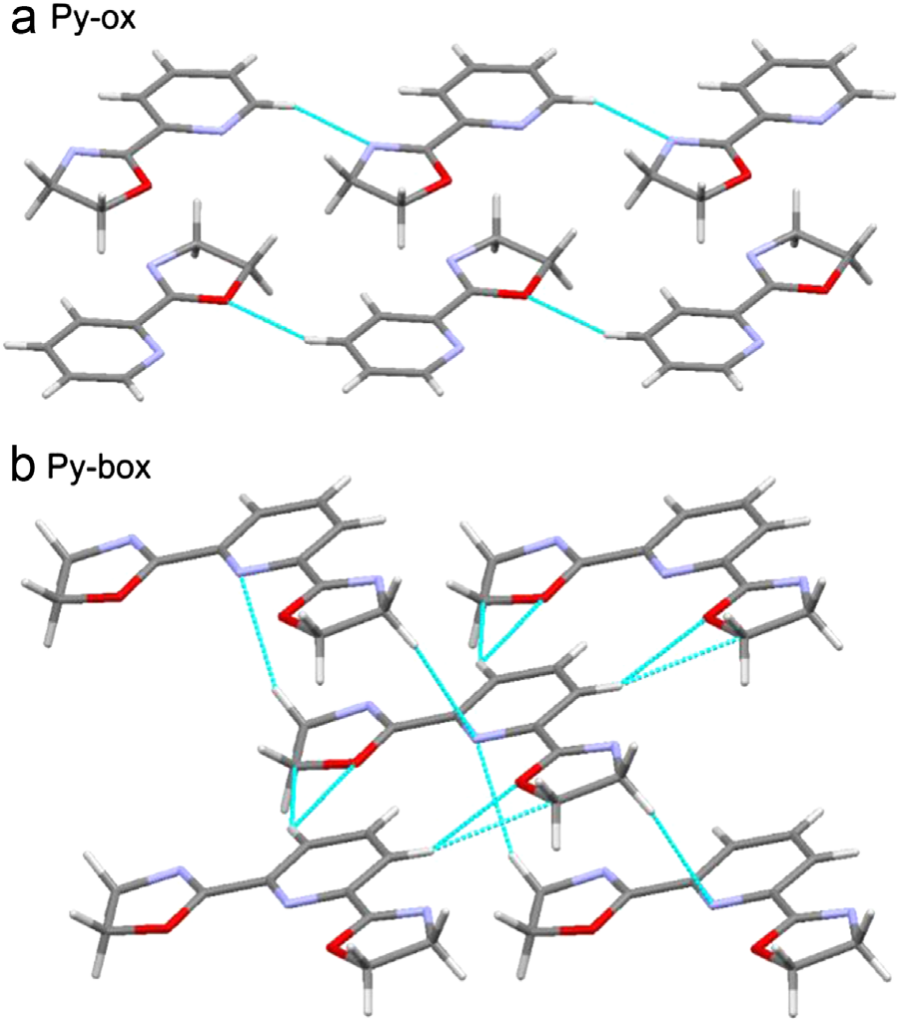
Association of molecule in the crystal structure. Hydrogen contacts are marked by dashed lines. The numbers of atoms and distances are omitted for clarity. All geometric parameters are in Table 2.

**Fig. 3:**
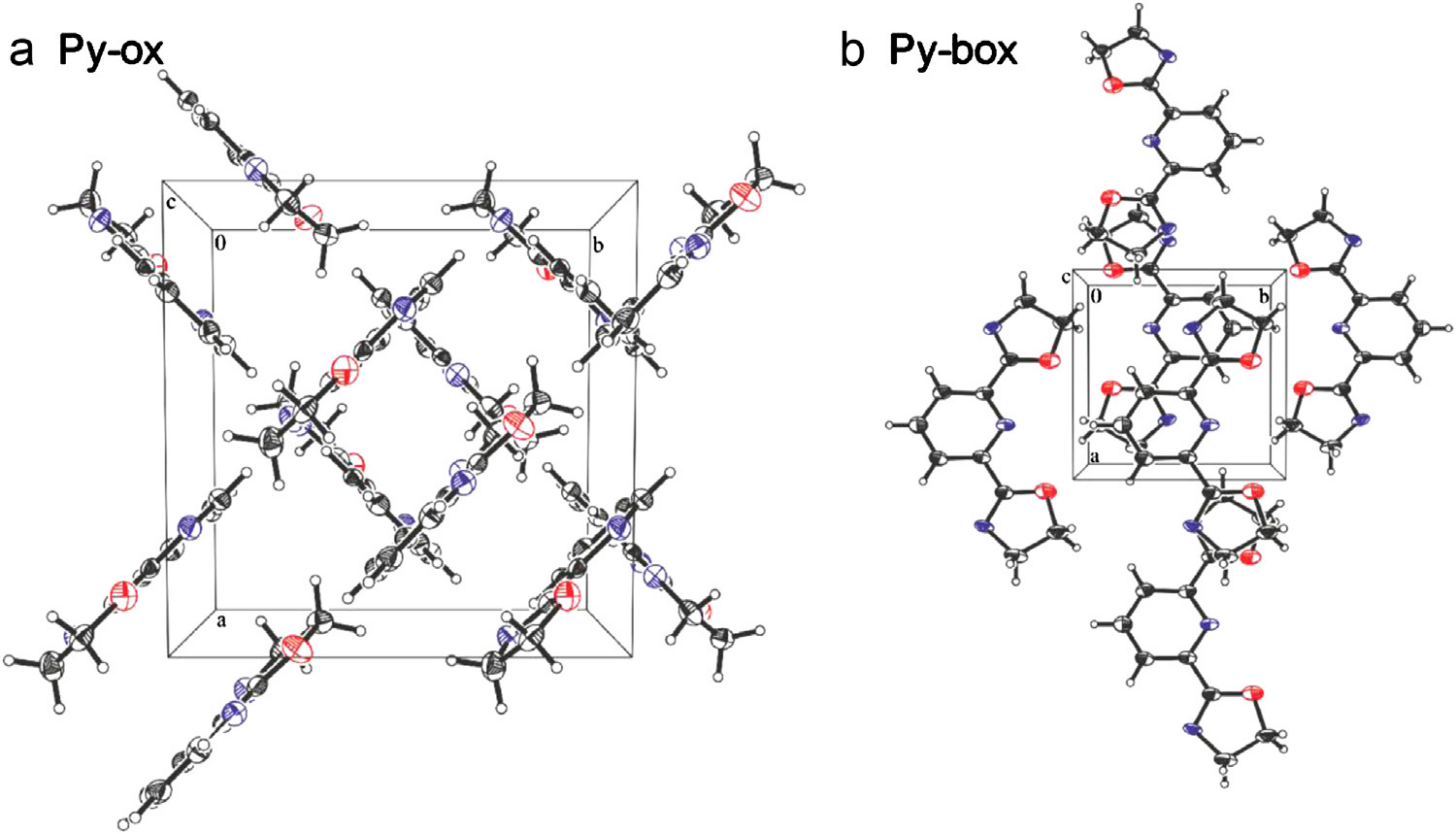
The crystal packing scheme of the title compounds. A view along the *c* axis of the crystals packing.

The single-crystal X-ray diffraction experiments were performed at 293.0(1)K on the Xcalibur diffractometer, equipped with a CCD area detector and a graphite monochromator for the MoKα radiation. The reciprocal space was explored by ω scans with detector positions at 60 mm distance from the crystal. The diffraction data processing of studied compounds (Lorentz and polarization corrections were applied) were performed using the CrysAlis CCD [21], [22]. Both structures Py-ox and Py-box were solved in the C2 and P2/n space group respectively, by direct methods and refined by a full-matrix least-squares method using SHELXL14 program [23], [24]. The H atoms were found based on geometrical parameters. In both structures H atoms were refined using a riding model. The structure drawings were prepared using SHELXTL and Mercury programs [25] (Fig. 1, Fig. 2, Fig. 3 and Table 1, Table 2).

**Table 1:** X-ray experimental details for 2-(1,3-oxazolin-2-yl)pyridine (Py-ox) and 2,6-bis(1,3-oxazolin-2-yl) pyridine (Py-box).

	**Py-ox**	**Py-box**
Chemical formula	C_8_H_8_N_2_O	C_11_H_11_N_3_O_2_
*M*_r_	148.16	217.23
Crystal system, space group	Monoclinic, *C*2	Monoclinic, *P*2/*n*
*a*, *b*, *c* (Å)	10.2571 (7), 10.0159 (6), 14.4647 (9)	6.4904 (8), 6.5835 (11), 11.9080 (19)
β (°)	97.497 (6)	94.215 (13)
*V* (Å^3^)	1473.31 (16)	507.45 (13)
*Z*	8	2
Measurement temperature	293.0(1)	293.0(1)
µ (mm^−1^)	0.09	0.10
Crystal size (mm)	0.4 × 0.3 × 0.2	0.5 × 0.4 × 0.3
Crystal colour	Colourless	
Crystal description	Plate	
Data collection		
Radiation wavelength	0.71073	
Radiation type	MoKα	
Source	fine-focus sealed tube	
Measurement device type	Xcalibur	
Detector area resolution	1024 × 1024 with blocks 2 × 2	
Absorption correction	–	
No. of measured, independent and observed [*I*>2σ(*I*)] reflections	5,034, 2786, 1587	3,172, 993, 459
*R*_int_	0.018	0.048
(sin θ/λ)_max_ (Å^−1^)	0.617	0.616
Refinement		
*R*[*F*^2^>2σ(*F*^2^)], *wR*(*F*^2^), *S*	0.030, 0.077, 0.86	0.057, 0.173, 0.87
No. of reflections	2786	993
No. of parameters	200	75
No. of restraints	1	0
Δρ_max_, Δρ_min_ (e Å^−3^)	0.11, −0.09	0.22, −0.18

**Table 2:** Selected geometric parameters (Å, °) for Py-ox and Py-box molecules.

**Structure 2 (Py-ox)**			
N1A-C2A	1.386 (7)	C5B-H5B	0.9300
N1A-C6A	1.394 (6)	C7A-N8A	1.292 (7)
C2A-C3A	1.367 (8)	C7A-O11A	1.314 (6)
C2A-H2A	0.9300	C6B-C7B	1.478 (7)
N1B-C6B	1.360 (6)	N8A-C9A	1.423 (7)
N1B-C2B	1.393 (7)	C7B-N8B	1.289 (6)
C3A-C4A	1.400 (8)	C7B-O11B	1.292 (7)
C3A-H3A	0.9300	C9A-C10A	1.518 (8)
C2B-C3B	1.344 (9)	C9A-H9AA	0.9700
C2B-H2B	0.9300	C9A-H9AB	0.9700
C4A-C5A	1.307 (8)	N8B-C9B	1.427 (7)
C4A-H4A	0.9300	C10A-O11A	1.470 (7)
C3B-C4B	1.361 (9)	C10A-H10A	0.9700
C3B-H3B	0.9300	C10A-H10B	0.9700
C5A-C6A	1.335 (6)	C9B-C10B	1.513 (8)
C5A-H5A	0.9300	C9B-H9BA	0.9700
C4B-C5B	1.345 (7)	C9B-H9BB	0.9700
C4B-H4B	0.9300	O11B-C10B	1.488 (6)
C6A-C7A	1.464 (7)	C10B-H10C	0.9700
C5B-C6B	1.342 (6)	C10B-H10D	0.9700
C2A-N1A-C6A	116.2 (5)	C5B-C6B-C7B	119.1 (5)
C3A-C2A-N1A	122.5 (6)	N1B-C6B-C7B	117.4 (5)
C3A-C2A-H2A	118.8	C7A-N8A-C9A	106.3 (5)
N1A-C2A-H2A	118.8	N8B-C7B-O11B	119.6 (6)
C6B-N1B-C2B	115.8 (5)	N8B-C7B-C6B	120.4 (6)
C2A-C3A-C4A	116.1 (6)	O11B-C7B-C6B	120.0 (5)
C2A-C3A-H3A	122.0	N8A-C9A-C10A	106.9 (6)
C4A-C3A-H3A	122.0	N8A-C9A-H9AA	110.3
C3B-C2B-N1B	121.2 (6)	C10A-C9A-H9AA	110.3
C3B-C2B-H2B	119.4	N8A-C9A-H9AB	110.3
N1B-C2B-H2B	119.4	C10A-C9A-H9AB	110.3
C5A-C4A-C3A	123.3 (6)	H9AA-C9A-H9AB	108.6
C5A-C4A-H4A	118.3	C7B-N8B-C9B	105.7 (5)
C3A-C4A-H4A	118.3	O11A-C10A-C9A	101.9 (4)
C2B-C3B-C4B	120.0 (6)	O11A-C10A-H10A	111.4
C2B-C3B-H3B	120.0	C9A-C10A-H10A	111.4
C4B-C3B-H3B	120.0	O11A-C10A-H10B	111.4
C4A-C5A-C6A	119.6 (6)	C9A-C10A-H10B	111.4
C4A-C5A-H5A	120.2	H10A-C10A-H10B	109.3
C6A-C5A-H5A	120.2	N8B-C9B-C10B	106.8 (4)
C5B-C4B-C3B	120.2 (6)	N8B-C9B-H9BA	110.4
C5B-C4B-H4B	119.9	C10B-C9B-H9BA	110.4
C3B-C4B-H4B	119.9	N8B-C9B-H9BB	110.4
C5A-C6A-N1A	122.4 (5)	C10B-C9B-H9BB	110.4
C5A-C6A-C7A	118.1 (5)	H9BA-C9B-H9BB	108.6
N1A-C6A-C7A	119.6 (5)	C7A-O11A-C10A	106.8 (5)
C6B-C5B-C4B	119.1 (5)	C7B-O11B-C10B	106.1 (5)
C6B-C5B-H5B	120.5	O11B-C10B-C9B	101.7 (5)
C4B-C5B-H5B	120.5	O11B-C10B-H10C	111.4
N8A-C7A-O11A	118.1 (5)	C9B-C10B-H10C	111.4
N8A-C7A-C6A	122.8 (5)	O11B-C10B-H10D	111.4
O11A-C7A-C6A	119.1 (6)	C9B-C10B-H10D	111.4
C5B-C6B-N1B	123.6 (5)	H10C-C10B-H10D	109.3

Symmetry code(s): (i) −*x*+1/2, *y*, −*z*+1/2.			

**Structure 1 (Py-Box)**			
N1-C2^i^	1.355 (3)	C5-O9	1.316 (4)
N1-C2	1.355 (3)	N6-C7	1.448 (4)
C2-C3	1.381 (4)	C7-C8	1.502 (4)
C2-C5	1.468 (4)	C7-H7A	0.9700
C3-C4	1.380 (4)	C7-H7B	0.9700
C3-H3	0.9300	C8-O9	1.471 (3)
C4-C3^i^	1.380 (4)	C8-H8A	0.9700
C4-H4	0.9300	C8-H8B	0.9700
C5-N6	1.293 (3)		
C2^i^-N1-C2	116.0 (4)	N6-C7-C8	105.1 (2)
N1-C2-C3	123.4 (3)	N6-C7-H7A	110.7
N1-C2-C5	116.5 (3)	C8-C7-H7A	110.7
C3-C2-C5	120.0 (2)	N6-C7-H7B	110.7
C4-C3-C2	119.5 (3)	C8-C7-H7B	110.7
C4-C3-H3	120.2	H7A-C7-H7B	108.8
C2-C3-H3	120.2	O9-C8-C7	104.0 (3)
C3^i^-C4-C3	118.1 (4)	O9-C8-H8A	110.9
C3^i^-C4-H4	121.0	C7-C8-H8A	110.9
C3-C4-H4	121.0	O9-C8-H8B	110.9
N6-C5-O9	118.1 (2)	C7-C8-H8B	110.9
N6-C5-C2	121.0 (3)	H8A-C8-H8B	109.0
O9-C5-C2	120.9 (2)	C5-O9-C8	105.8 (2)
C5-N6-C7	106.9 (2)		

#### 2.3.1 2-(1,3-oxazolin-2-yl)pyridine (Py-ox)







#### 2.3.2 2,6-bis(1,3-oxazolin-2-yl)pyridine (Py-box)







### 2.4 NMR

**Fig. 4:**
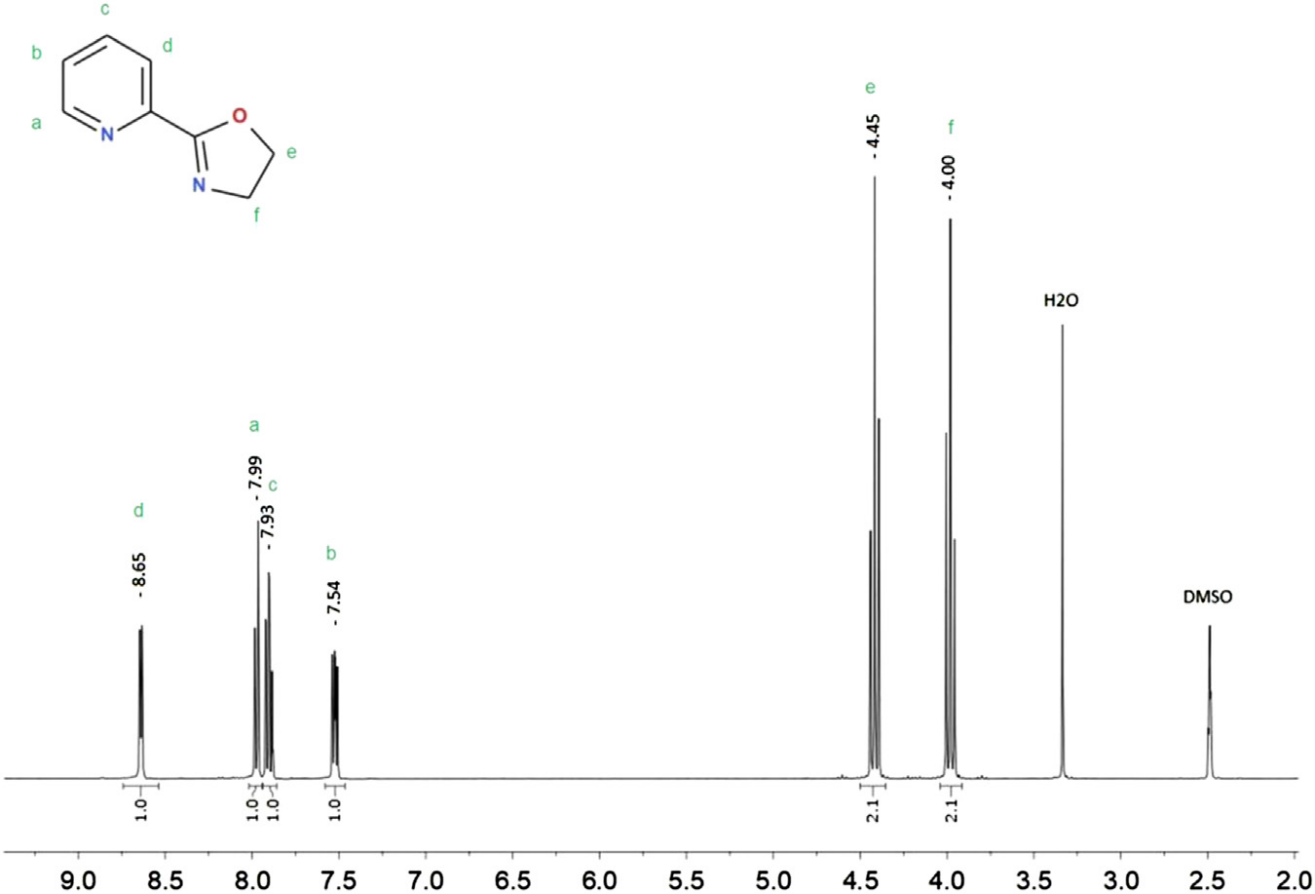
^1^H NMR spectrum for 2-(1,3-oxazolin-2-yl)pyridine (Py-ox) in DMSO-*d6*.

**Fig. 5:**
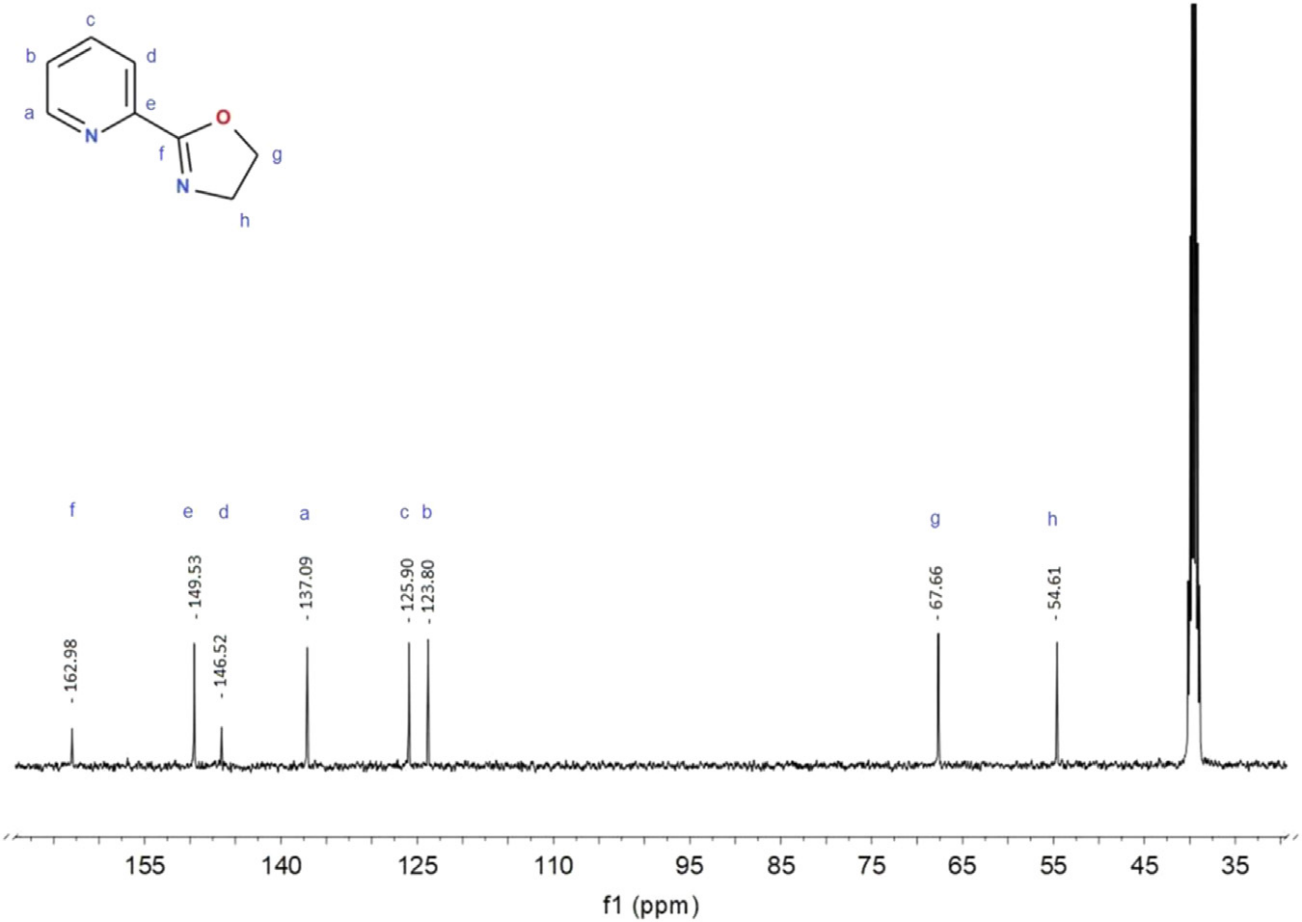
^13^C NMR spectrum for 2-(1,3-oxazolin-2-yl)pyridine (Py-ox) in DMSO-*d6*.

**Fig. 6:**
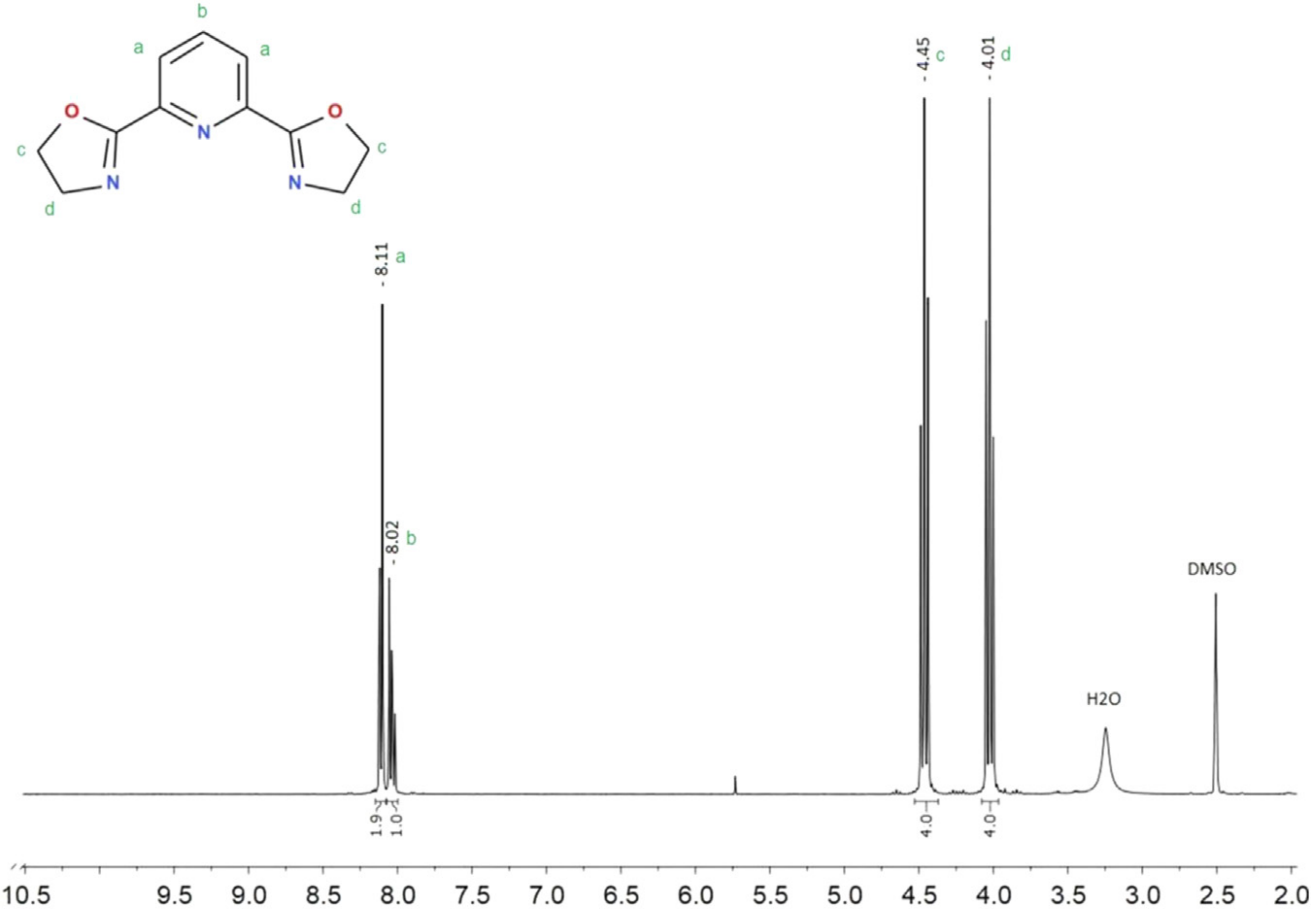
^1^H NMR spectrum for 2,6-bis(1,3-oxazolin-2-yl)pyridine (Py-box) in DMSO-*d6*.

**Fig. 7:**
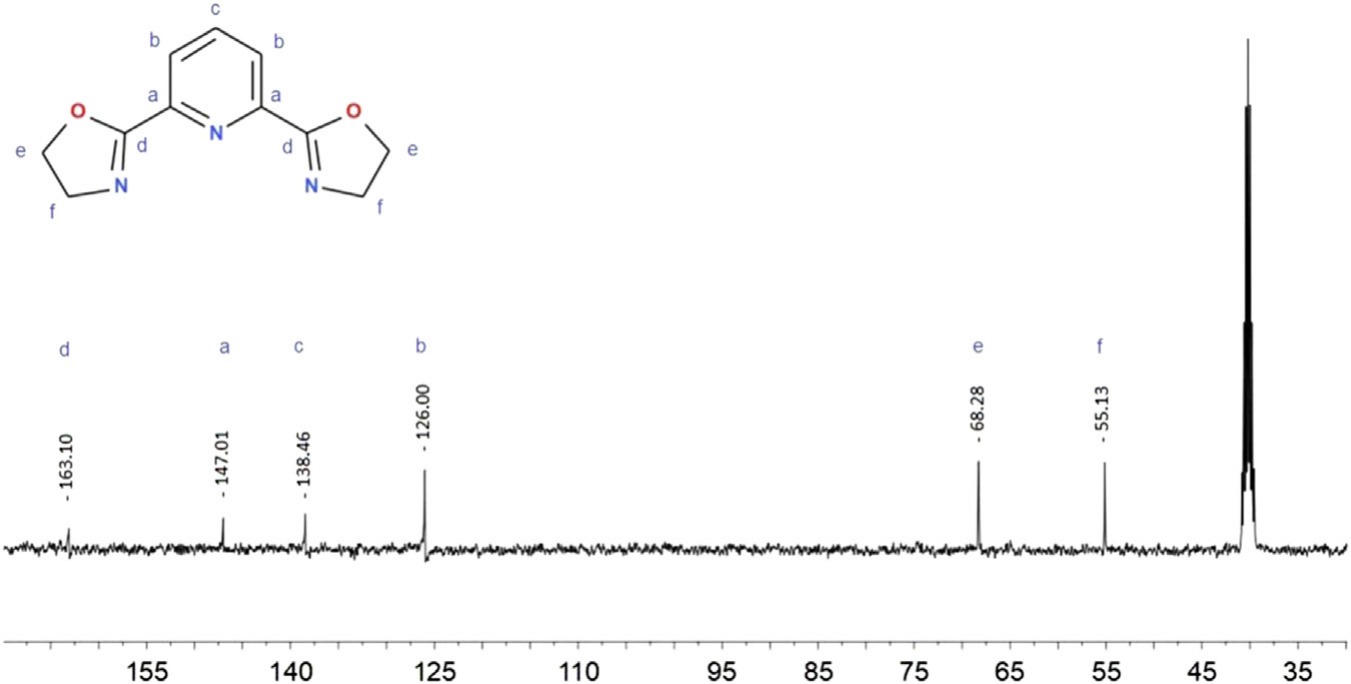
^13^C NMR spectrum for 2,6-bis(1,3-oxazolin-2-yl)pyridine (Py-box) in DMSO-*d6*.

Bruker Ultrashield spectrometer 400 MHz, solvent DMSO-d6, TMS standard. Concentration: 15 mg in 0.6 ml (Fig. 4, Fig. 5, Fig. 6, Fig. 7).

### 2.5 GC–MS

**Fig. 8:**
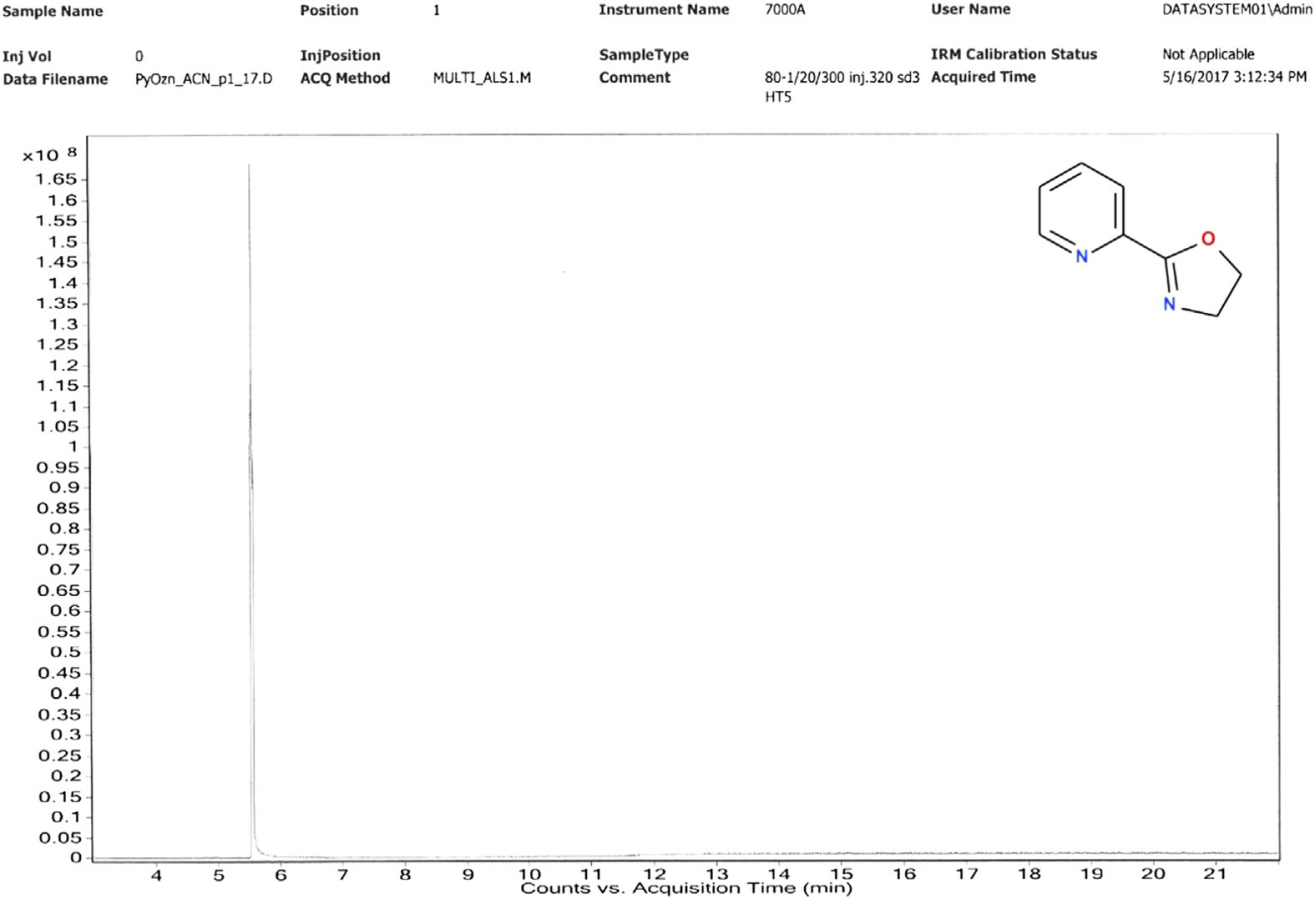
GC analysis of 2-(1,3-oxazolin-2-yl)pyridine (Py-ox).

**Fig. 9:**
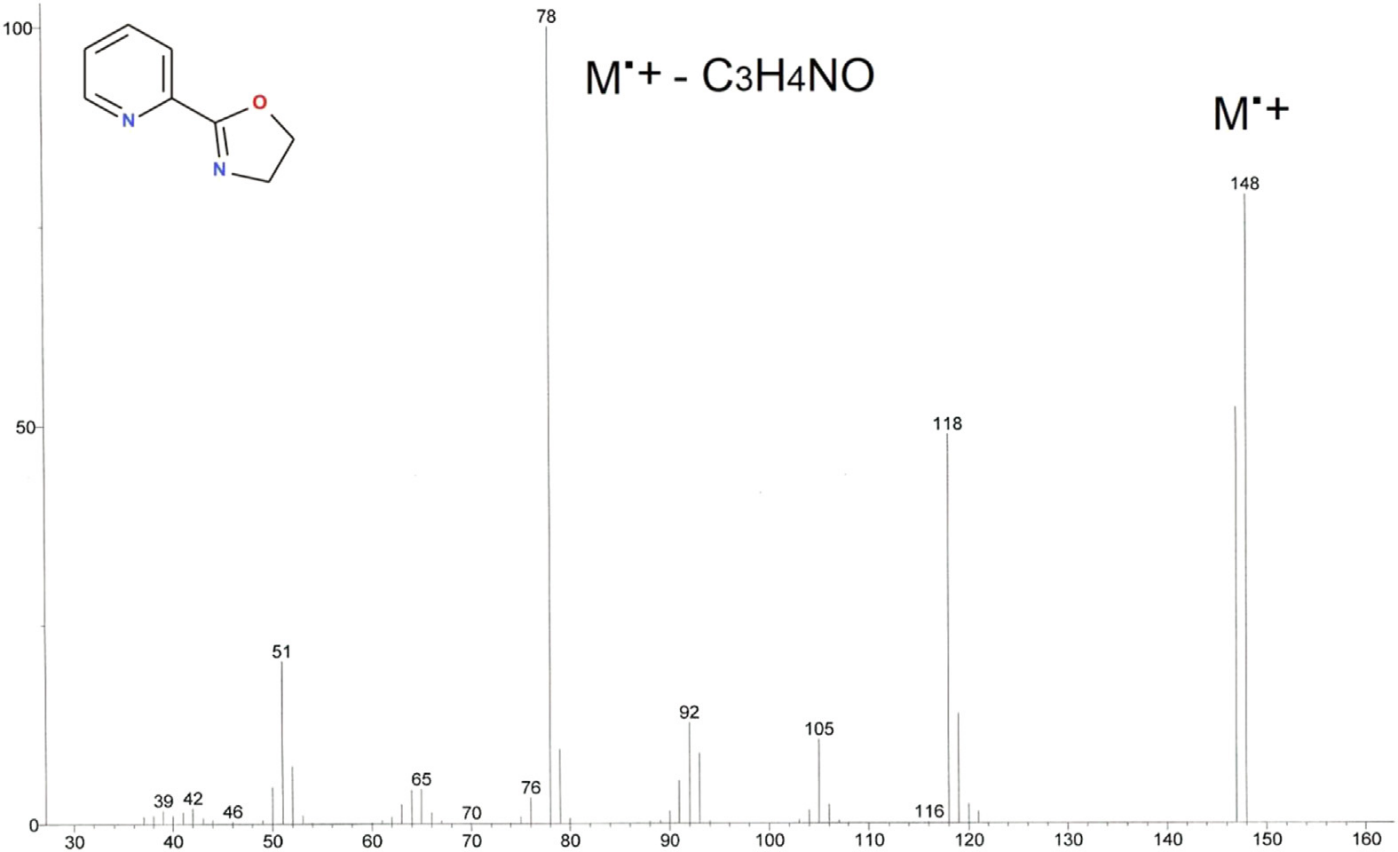
MS analysis of 2-(1,3-oxazolin-2-yl)pyridine (Py-ox).

**Fig. 10:**
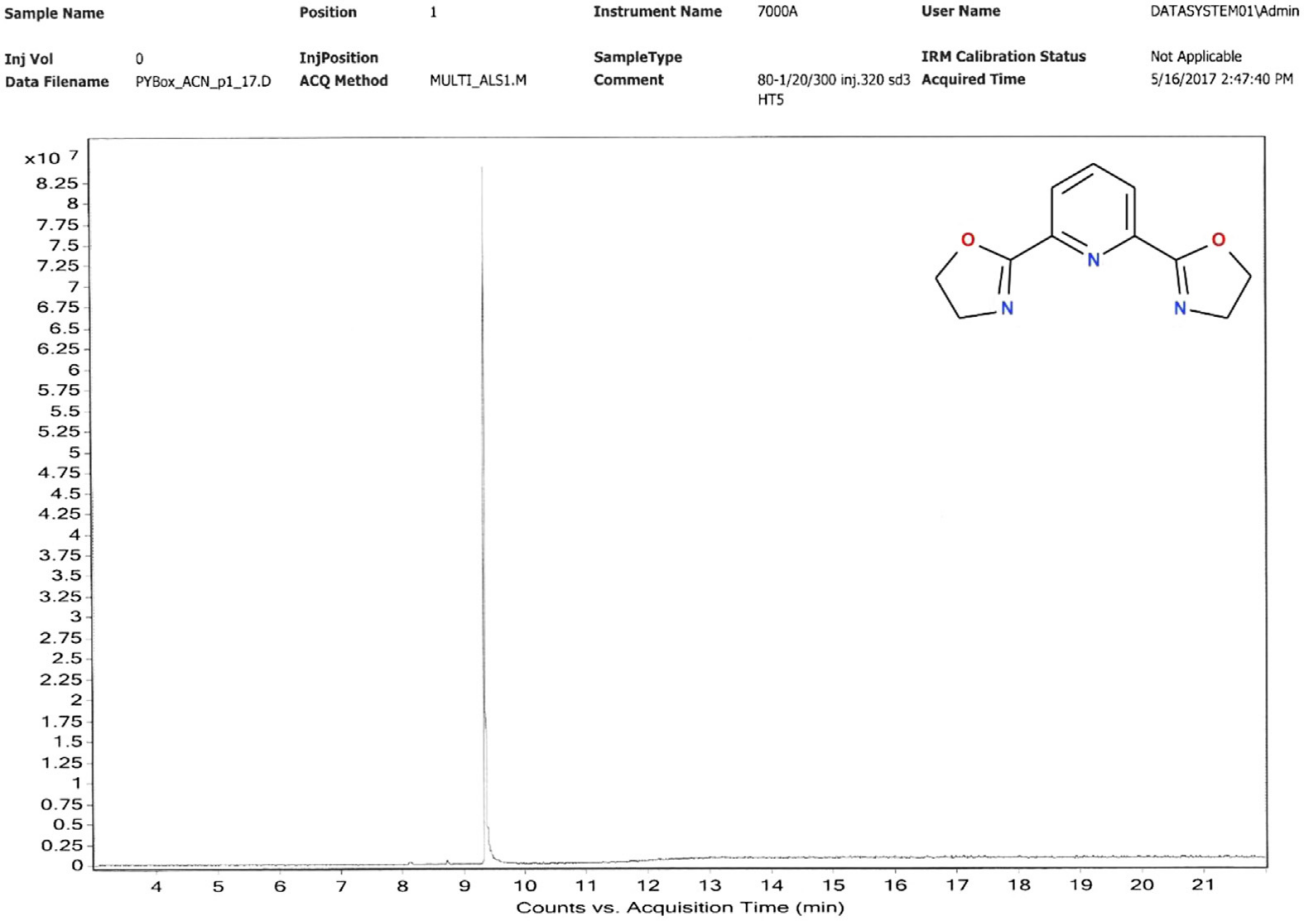
GC analysis of 2,6-bis(1,3-oxazolin-2-yl)pyridine (Py-box).

**Fig. 11:**
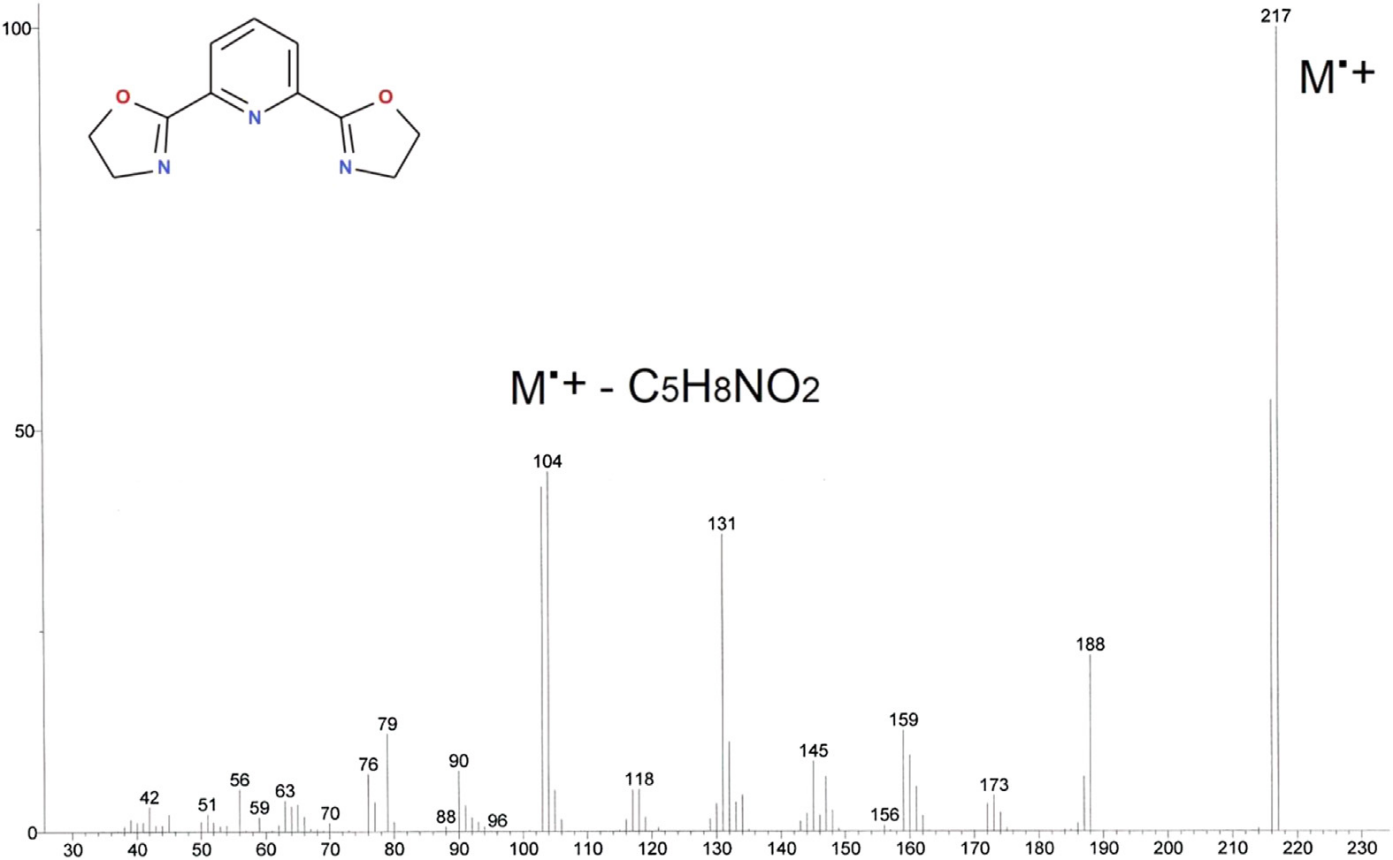
MS analysis of 2,6-bis(1,3-oxazolin-2-yl)pyridine (Py-box).

Hewlett Packard HP7890 A GC system, equipped with 7000 GC/MS triple-quadrupol and HP-5 capilar 300 m × 0.32 mm column with 0.25 µm dimethylpolysilloxane stationary phase, dopped by 5% of phenylpolysilloxane (Fig. 8, Fig. 9, Fig. 10, Fig. 11).

### 2.6 DSC

**Fig. 12:**
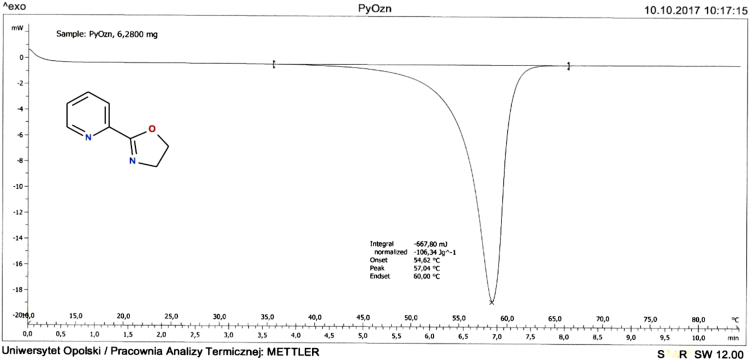
DSC analysis of 2-(1,3-oxazolin-2-yl)pyridine (Py-ox).

**Fig. 13:**
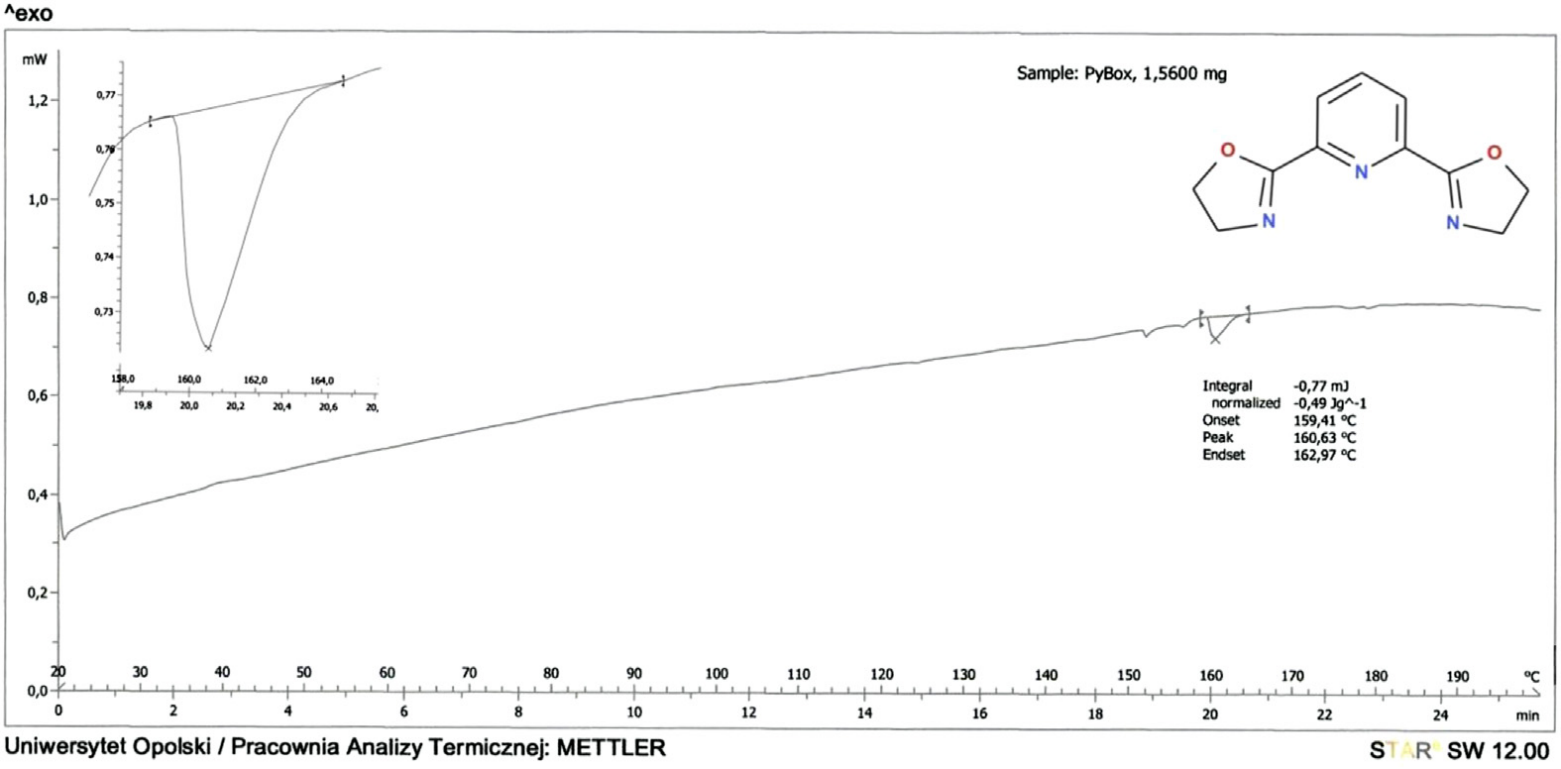
DSC analysis of 2,6-bis(1,3-oxazolin-2-yl)pyridine (Py-box).

The melting temperatures were measured by differential scanning calorimetry DSC 2010 TA instrument calorimeter equipped with an automated sampler. The data were collected with the heat/cool/heat cycle at a heating rate of 10 °C/min under a nitrogen atmosphere (Figs. 12 and 13).
