# Supplementary material for: 2-(1,3-Oxazolin-2-yl)pyridine and 2,6-bis(1,3-oxazolin-2-yl) pyridine
Source: Data Brief. 2018 Oct 4;21:449–65. doi: 10.1016/j.dib.2018.09.129 (PMC6198059; doi:10.1016/j.dib.2018.09.129)
Supplement: Supplementary file 1 — Supplementary material. [file mmc1.pdf]

Conflicts of Interest Statement

Manuscript title: **2-(1,3-Oxazolin-2-yl)pyridine and 2,6-bis(1,3-oxazolin-2-yl)pyridine**

Submission: **Data in Brief**

On behalf of all authors, I hereby declare that there is NO affiliations with or involvement in any organization or entity with any financial interest (such as honoraria; educational grants; participation in speakers' bureaus; membership, employment, consultancies, stock ownership, or other equity interest; and expert testimony or patent-licensing arrangements), or non-financial interest (such as personal or professional relationships, affiliations, knowledge or beliefs) in the subject matter or materials discussed in this manuscript.

Corresponding author name: Wioletta Ochędzan-Siodłak

26-07-2018 Wioletta Ochędzan-Siodłak
